# Primary anterior cruciate ligament repair—morphological and quantitative assessment by 7-T MRI and clinical outcome after 1.5 years

**DOI:** 10.1007/s00330-024-10603-z

**Published:** 2024-02-12

**Authors:** Milena L. Pachowsky, Stefan Söllner, Kolja Gelse, Jannik Sambale, Armin M. Nagel, Georg Schett, Marc Saake, Michael Uder, Frank W. Roemer, Rafael Heiss

**Affiliations:** 1 Department of Internal Medicine 3 – Rheumatology and Immunology, University Hospital Erlangen, Friedrich-Alexander-Universität Erlangen-Nürnberg, Ulmenweg 18, 91054 Erlangen, Germany; 2 Department of Orthopaedic and Trauma Surgery, Waldkrankenhaus St. Marien, Erlangen, Germany; 3 Department of Trauma and Orthopaedic Surgery, University Hospital Erlangen, Friedrich-Alexander-Universität Erlangen-Nürnberg, Erlangen, Germany; 4 Department of Trauma and Orthopaedic Surgery, Klinikum Traunstein, Traunstein, Germany; 5 Friedrich-Alexander-Universität Erlangen-Nürnberg, Erlangen, Germany; 6 Institute of Radiology, University Hospital Erlangen, Friedrich-Alexander-Universität Erlangen-Nürnberg, Erlangen, Germany; 7 Division of Medical Physics in Radiology, German Cancer Research Center (DKFZ), Heidelberg, Germany; 8 Quantitative Imaging Center (QIC), School of Medicine, Boston University, Chobanian & Avedisian, Boston, MA USA

**Keywords:** Magnetic resonance imaging, Knee, Ligaments, Cartilage, Orthopedics

## Abstract

**Objectives:**

The purpose of this study was to assess morphological and quantitative changes of the anterior cruciate ligament (ACL) and cartilage after ACL repair.

**Methods:**

7T MRI of the knee was acquired in 31 patients 1.5 years after ACL repair and in 13 controls. Proton density-weighted images with fat saturation (PD-fs) were acquired to assess ACL width, signal intensity, elongation, and fraying. T2/T2* mapping was performed for assessment of ACL and cartilage. Segmentation of the ACL, femoral, and tibial cartilage was carried out at 12 ROIs. The outcome evaluation consisted of the Lysholm Knee Score and International Knee Documentation Committee (IKDC) subjective score and clinical examination.

**Results:**

ACL showed a normal signal intensity in 96.8% and an increased width in 76.5% after repair. Fraying occurred in 22.6% without having an impact on the clinical outcome (Lysholm score: 90.39 ± 9.75, *p *= 0.76 compared to controls). T2 analysis of the ACL revealed no difference between patients and controls (*p *= 0.74). Compared to controls, assessment of the femoral and tibial cartilage showed a significant increase of T2* times in all ROIs, except at the posterolateral femur. Patients presented a good outcome in clinical examination with a Lysholm score of 87.19 ± 14.89 and IKDC of 80.23 ± 16.84.

**Conclusion:**

T2 mapping results suggest that the tissue composition of the ACL after repair is similar to that of a native ACL after surgery, whereas the ACL exhibits an increased width. Fraying of the ACL can occur without having any impact on functional outcomes. T2* analysis revealed early degradation at the cartilage.

**Clinical relevance statement:**

MRI represents a noninvasive diagnostic tool for the morphological and compositional assessment of the anterior cruciate ligament after repair, whereas knowledge about post-surgical alterations is crucial for adequate imaging interpretation.

**Key Points:**

*• There has been renewed interest in repairing the anterior cruciate ligament with a proximally torn ligament.*

*• T2 times of the anterior cruciate ligament do not differ between anterior cruciate ligament repair patients and controls.*

*• T2 mapping may serve as a surrogate for the evaluation of the anterior cruciate ligament after repair.*

## Introduction

One of the most common knee injuries among adolescent and mid-aged athletes is the tear of the anterior cruciate ligament (ACL) [1]. Reconstruction of the ACL with hamstring or patellar tendon autografts has been the standard of care in surgical treatments of ACL tears for many years [2, 3]. The dogma that the ACL cannot heal has been called into question during the last years, and it was shown that certain tears may have a healing capacity and exhibit better clinical outcomes compared to non-healed ACLs [4–6]. Hence, ACL repair techniques have made a resurgence in recent years. However, the topic raises diverging opinions among orthopedic surgeons and leads to discussions about the benefit of restoring native anatomic features versus the risks of healing failure and re-rupture. Preserving the native tissue and proprioception [7, 8], the potentially decreased postoperative pain and increased range of motion have been described as important advantages [9]. Recent and historical study results suggest that primary ACL repair (meaning the preservation of the tissue) is reserved for proximal tears due to better vascularity [10] and good healing potential [11].

The time required to complete the remodeling process and the imaging appearance in MRI are unknown for ACL repair. Imaging methods help surgeons and patients to gather information about the status of the injured and treated joint. Magnetic resonance imaging (MRI) is the most widely used imaging technique for monitoring ACL grafts after surgical reconstruction [12–14]. High-field MRI at 3 Tesla (T) and, in particular, ultrahigh-field MRI at 7 T suggests better visualization of small anatomical structures and give better diagnostic performance [15]. The signal-to-noise ratio (SNR) typically increases at least linearly with magnetic field strength [15, 16]. This gain might be used to increase spatial resolution and for a better visualization of the microstructure of ACL repair during follow-up examinations [17]. Besides morphological imaging, compositional MRI techniques, including T2/T2* mapping, are potential candidates to assess ACL remodeling and cartilage [18–22]. It allows a noninvasive quantification of tissues’ water content and assessment of its anisotropy, thereby providing a proxy of structural changes in the collagen matrix [22]. Previous research established quantitative MRI mapping techniques as noninvasive, reproducible, and reliable biomarkers to evaluate connective tissues [23].

The objective of this study was to perform an analysis of the morphological imaging appearance of the ACL after primary repair at a single center and to assess the structural properties of the ACL repair tissue and also the cartilage of the knee 1.5 years after surgery using high resolution and compositional MRI at 7 T. Additionally, we aimed to assess the clinical outcome of the patients after primary ACL repair.

## Material and methods

For this study, ethics approval was granted by the Clinical Ethics Committee of the local university. Written informed consent was obtained from all participating patients and healthy controls.

### Patients and healthy controls

All consecutive patients who underwent arthroscopic repair of the ACL between 01/2018–06/2018 were enrolled in the study and selected for a prospective investigation. The participants of the control group were age-matched and had no history of knee injury and no incidental findings in the MRI. Inclusion criteria for the patient group were: unilateral acute proximal ACL rupture, no previous knee ligament surgery, and absence of ligament injury of the contralateral knee. Preoperative magnetic resonance imaging confirming a proximal avulsion tear of the ACL was required. Exclusion criteria were the presence of grade 3 or 4 cartilage degeneration both on MRI and in arthroscopy and intraoperative insufficient distal remnant length (leading to ACL repair with a tendon graft). The arthroscopic primary ACL was performed within 2–3 weeks after injury and followed a standardized surgical approach.

### ACL repair surgical technique

All surgeries followed a structured protocol. A standardized arthroscopic surgical approach was performed with two standard portals (anterolateral and anteromedial). The ACL had to have a sufficient distal remnant length to reapproximate it to the femoral footprint. In brief, the remnant was sutured with the scorpion needle using a non-resorbable suture (No. 2 FiberWire sutures (Arthrex)). For the transosseous button fixation, a femoral tunnel was drilled from the insertion site to the lateral femoral cortex. Button and sutures were then carried through the hole, and the button was flipped on the lateral femoral condyle. Sutures were pulled through the button. All patients received additional internal bracing (FiberTape, Arthrex) with ACL repair. This technique reinforces the ligament as a secondary stabilizer by protecting it during the healing phase [24]. The ligament was then reapproximated to its footprint by tightening the sutures. Through a third skin incision over the medial tibia head, a second drill hole was made (between the anteromedial tibia cortex and the anterior part of the ACL tibia insertion). The tape was channeled distally along the ligament, through the drill hole, and exits at the anteromedial cortex of the tibia, where it was tied over a button. The FiberTape forms an adjustable loop through the femoral button and the tape was tensioned in full extension of the knee.

### Clinical follow-up investigation

The follow-up investigation was scheduled 1.5 years after the surgery. Data for the evaluation were collected in a clinical examination and by means of a survey on subjective well-being and fitness. Patient-reported outcome scores were documented, consisting of the Lysholm Knee Score [25] and IKDC Subjective Score [26]. The clinical follow-up examination of the patients included effusion, range of motion (ROM), ligament stability, and the IKDC score.

### Imaging follow-up investigation

7 T MRI was performed 1.5 years after the surgery in all patients using a standardized MRI protocol. Age-matched healthy controls were examined with the same protocol.

## Image acquisition

MRI was performed on a 7 T MRI (MAGNETOM Terra, Siemens Healthineers) in combination with a dedicated 1-channel transmit/ 28-channel receive knee array coil (Quality Electrodynamics). Five days prior to the MRI, daily routine activities were allowed, but not sports. Patients were in a supine position with the knee tightly fixed and fully extended in a neutral rotation position in the center of the coil. Proton-density turbo spin echo sequences with fat saturation (PD-fs) in three planes (transversal, coronal, sagittal) were acquired for the morphological assessment. T2 mapping for the quantitative evaluation of the ACL was performed using a parasagittal plane, providing in-plane images of the entire course of the ACL. Sagittal T2* mapping for the assessment of the femoral and tibial cartilage was acquired (Table 1).

**Table 1:** Acquisition parameters of 7T knee MRI

Sequences	PD tse fs sag	PD tse fs cor	PD tse fs tra	T2 mapping para-sag	T2* mapping sag
Time of repetition (ms)	4800.0	4400.0	4400.0	2500.0	20.0
Time of echo (ms)	38.0	38.0	38.0	14.7, 29.4, 44.1, 58.8, 73.5, 88.2	2.68, 5.07, 7.46, 9.85, 12.24, 14.63
Field of view (mm)	160	160	160	160	160
Voxel size (mm)	0.2 x 0.2 x 2.5	0.2 x 0.2 x 2.5	0.2 x 0.2 x 2.5	0.4 x 0.4 x 2.0	0.5 x 0.5 x 0.5
Acquisition time (min)	6:54	3:20	3:20	4:19	6:10

## Image analysis

### Morphological MRI assessment

All 7 T images were assessed with regard to width, continuity, elongation, and signal intensity of the ACL repair by one radiologist and one orthopedic surgeon, both with special interest in musculoskeletal MRI. The reading was performed together in consensus. After 6 months, the reading was repeated separately by both to obtain inter- and intra-observer reliability.

The width of the ACL was assessed in the sagittal, coronal, and transversal planes and graded according to the following subjective subcategories: normal, increased, and decreased. The widest diameter of the ACL was also measured in the sagittal images.

The continuity of the ACL was evaluated in the sagittal plane with the following gradation: continuous, fraying, and non-continuous. Fraying describes the loosening of ACL tissue with the bulging of ACL fibers into the knee joint. We compared Lysholm scores from patients with fraying with those patients whose ACL tissue had a continuous appearance to determine whether fraying had an effect on the clinical outcome.

The signal intensity of the ACL was assessed according to the following aspects: Homogenous hyperintense and normal heterogeneous appearance [27]. Signal intensity was assessed in both sagittal and coronal planes. In addition, the ACL was judged for the presence of elongation, meaning a subjective curved prolongation of the ligaments’ course.

## Quantitative MRI assessment

For the compositional analysis of the ACL and the articular cartilage T2 maps, respectively T2* maps, were used. The MRI data were transferred to a Syngo (Leonardo) workstation (Siemens Healthineers). T2/T2* maps were obtained using a pixel-wise, mono-exponential, non-negative least-squares-fit analysis. The parallel analysis of morphological and quantitative series allowed for direct comparison and co-registration of anatomic and compositional data sets.

**Fig. 1:**
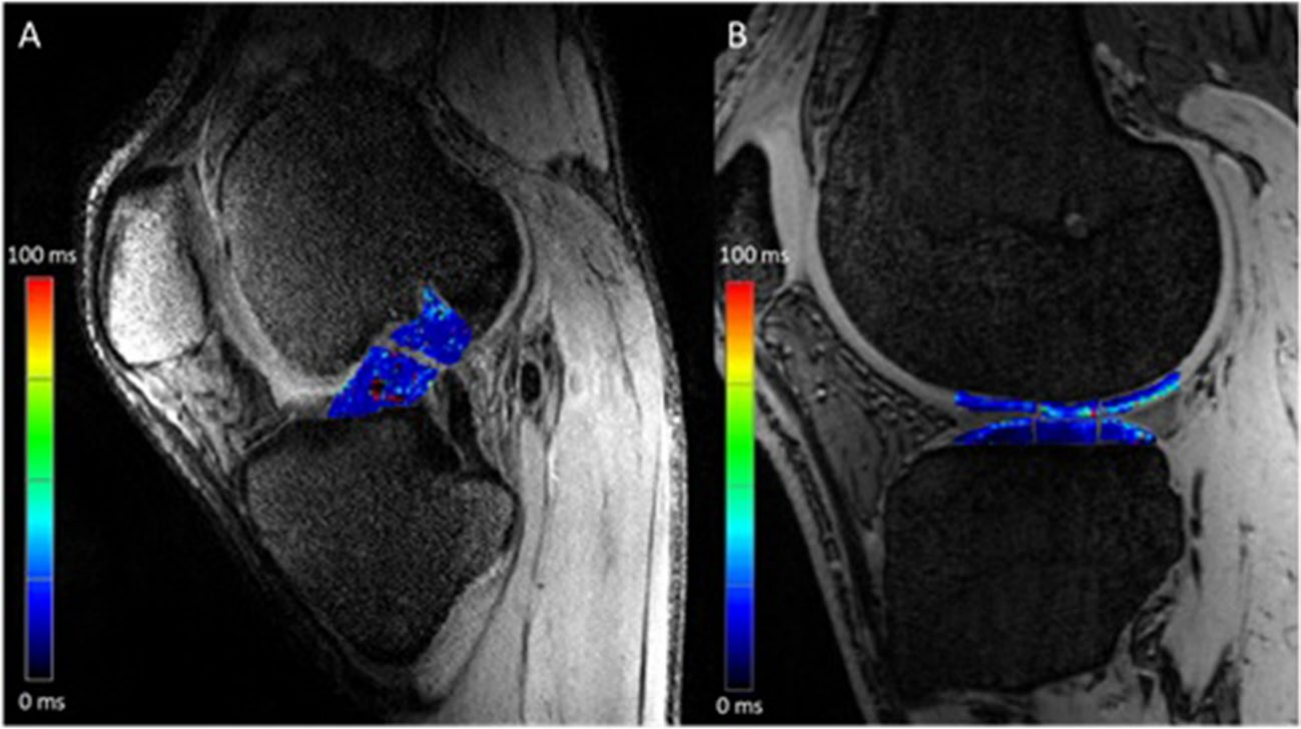
**A** 7T MRI image of the first echo of parasagittal T2 mapping with merged regions of interest of the corresponding T2 map for the anterior cruciate ligament of a 26-year-old female patient after ACL repair. **B** Image of the first echo of sagittal T2* mapping with merged regions of interest of the corresponding T2* map for the tibial and femoral cartilage

The ACL tissue was divided into 2 ROIs (cranial and caudal) for calculating the average T2 values derived from the T2 maps of the base of the ACL and the former tip of the remnant, see Fig. 1.

The T2* maps of the cartilage were analyzed using a region-of-interest analysis (ROI) according to previous recommendations [28]. ROI analysis was performed for the tibial and femoral cartilage surfaces of the knee joint. The ROIs were applied manually, and the mean T2* values for each ROI were calculated automatically. Three regions of interest (anterior, central, and posterior) were placed, each at the medial and lateral aspect of the tibia and femur, ending up with 12 regions in total for cartilage assessment (Fig. 1). Two neighboring slices at each anatomic location were segmented to reduce partial volume effects.

After a delay of 6 months, quantitative measurements were repeated to obtain reliability.

## Statistical analysis

Statistical analyses were performed using the SPSS 24.0 software (IBM Corporation). Continuous variables are displayed with means±standard deviations (SDs). Nominal variables are described with frequencies (%). In the first step of statistical analysis, the group of patients with concomitant injuries was compared to those with isolated ACL rupture. Due to no relevant differences, we pooled the data of these groups.

Results of patients and the control group were compared, using independent t tests for continuous variables and chi-squared tests or Fisher exact tests (for samples with a value < 5 in the contingency table) for nominal data. All tests were two-sided, and a difference of *p* < 0.05 was considered statistically significant.

The inter- and intrareader reproducibility were evaluated using (i) Cohen Kappa for nominal categorical variables (signal intensity and elongation) and (ii) weighted Kappa for ordered categorical variables (width and continuity). Kappa values (*K*) of 0.41–0.60, 0.61–0.80, and 0.81–1.0 are considered to indicate moderate, substantial, and almost perfect agreement, respectively. The reliability of T2 analysis of the ACL repair and T2* of the cartilage was evaluated by using the intraclass correlation coefficient (ICC). The ICC values were graded as follows: excellent reliability (0.75 > ICC ≤ 1), fair to good reliability (0.4 ≥ ICC ≤ 0.75), and poor reliability (0 ≥ ICC < 0.4).

## Results

### Study population

In total, 31 patients with ACL repair were enrolled in the study, and 13 healthy controls were without any history of knee injury. Demographic data are displayed in Table 2.

**Table 2:** Demographic data of patients and controls

Demographic data	Bracing	Control	
*n*	31	13	
	Mean ± SD	Mean ± SD	*p* value difference
Age (years)	28.79 ± 8.26	28.31 ± 2.84	n.s.
Weight (kg)	79.31 ±10.94	76.46 ± 12.89	n.s.
Height (cm)	1.76 ± 0.08	1.78 ± 9.13	n.s.
BMI (kg/m^2^)	25.33 ± 2.91	23.85 ± 2.56	n.s.
Gender (male vs. female %)	64.5 vs. 35.5	61.5 vs. 38.5	n.s.

Seven patients presented with focal cartilage defects on MRI, with four patients having Outerbridge grade 1 and 3 patients with grade 2 lesions. None of these cartilage defects was placed at the anatomic locations of quantitative T2* cartilage assessment. No further pathologies, such as meniscal tears or accompanying injuries of ligaments other than the ACL, were detected. In the MRI of healthy controls, no pathologies at the knee joint were present.

### Clinical assessment

The interval between injury of the knee and surgery was 14.35 ± 7.73 days. Acquisition of the MRI and clinical assessment was performed with a mean follow-up time of 18.29 ± 6.49 months after the surgery. The patient-reported outcomes of the entire ACL repair cohort were a mean Lysholm score of 87.19 ± 14.89 and a mean IKDC score of 80.23 ± 16.84.

ROM assessment showed on average, maximal extension/flexion of 4/0/136 degrees of the patients’ knees on the operated side and 5/0/142 on the non-operated side.

The majority of the patients (26, 83.87%) with ACL repair showed no effusion, three patients (9.68%) showed mild effusion, one patient had moderate effusion, and one patient had severe effusion.

The manual Lachman test was assessed as normal for 12 of the patients (38.71%), as nearly normal for 17 of the patients (54.84%), and as abnormal for two patients (6.5%).

One patient reported a failure after ACL repair in the sense of a re-rupture, which resulted in a failure rate of 3% in our patient population.

### Image analysis

#### Morphological MRI assessment of the ACL repair

**Fig. 2:**
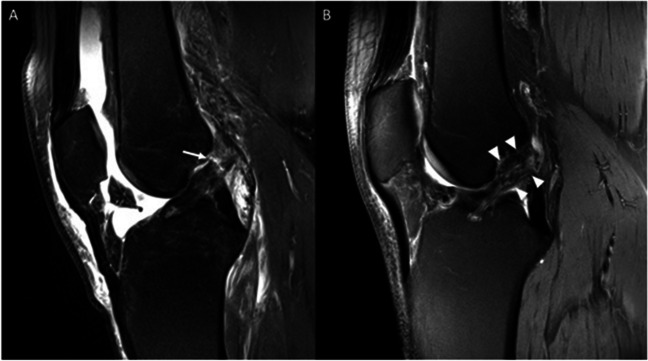
**A** 1.5T proton density-weighted fat-saturated MRI image of a 23-year-old male patient with a tear of the proximal ACL (arrow) after knee distortion. **B** 7T proton density-weighted fat-saturated MRI image of the same patient 1.5 years after primary ACL repair. The ACL appears continuous with an increased width in the proximal portion (arrowheads)

The width of the ACL repair was assessed as normal in the majority of the coronal slices (82.4 %), as normal in 11.8% of the cases in the sagittal plane, and in 17.6 % of the transversal views. Increased width of the ACL after repair was described in the sagittal (76.5%) and transversal (70.6%) plane (Fig. 2). In the control group, all of the ACLs were assessed as normal in all planes, see Table 3, section A. The width of the reattached ACLs was 1.04 ± 0.23 cm, and the ACLs of controls had an average width of 0.87 ± 0.15 cm (*p *= 0.026).

**Table 3:** Morphological assessment of the ACL in patients and controls, A width, B continuity, C signal intensity

A: Width of ACL	Patients	Controls
Sagittal		
Normal	4 (11.8%)	13 (100%)
Increased	26 (76.5%)	0 (0%)
Decreased	1 (2.9%)	0 (0%)
Coronal		
Normal	28 (82.4%)	13 (100%)
Increased	2 (5.9%)	0 (0%)
Decreased	1 (2.9%)	0 (0%)
Transversal		
Normal	6 (17.6%)	13 (100%)
Increased	24 (70.6%)	0 (0%)
Decreased	1 (2.9%)	0 (0%)
B: ACL continuity		
Continuous	23 (74.2%)	13 (100%)
Fraying	7 (22.6%)	0 (0%)
Non-continuous	1 (3.2%)	0 (0%)
C: ACL signal intensity		
Sagittal		
Homogenous hyperintense	1 (3.2%)	0 (0%)
Normal signal intensity	30 (96.8%)	13 (100%)
Coronal		
Homogenous hyperintense	1 (3.2%)	0 (0%)
Normal signal intensity	30 (96.8%)	13 (100%)

#### Continuity and signal intensity of the ACL repair

**Fig. 3:**
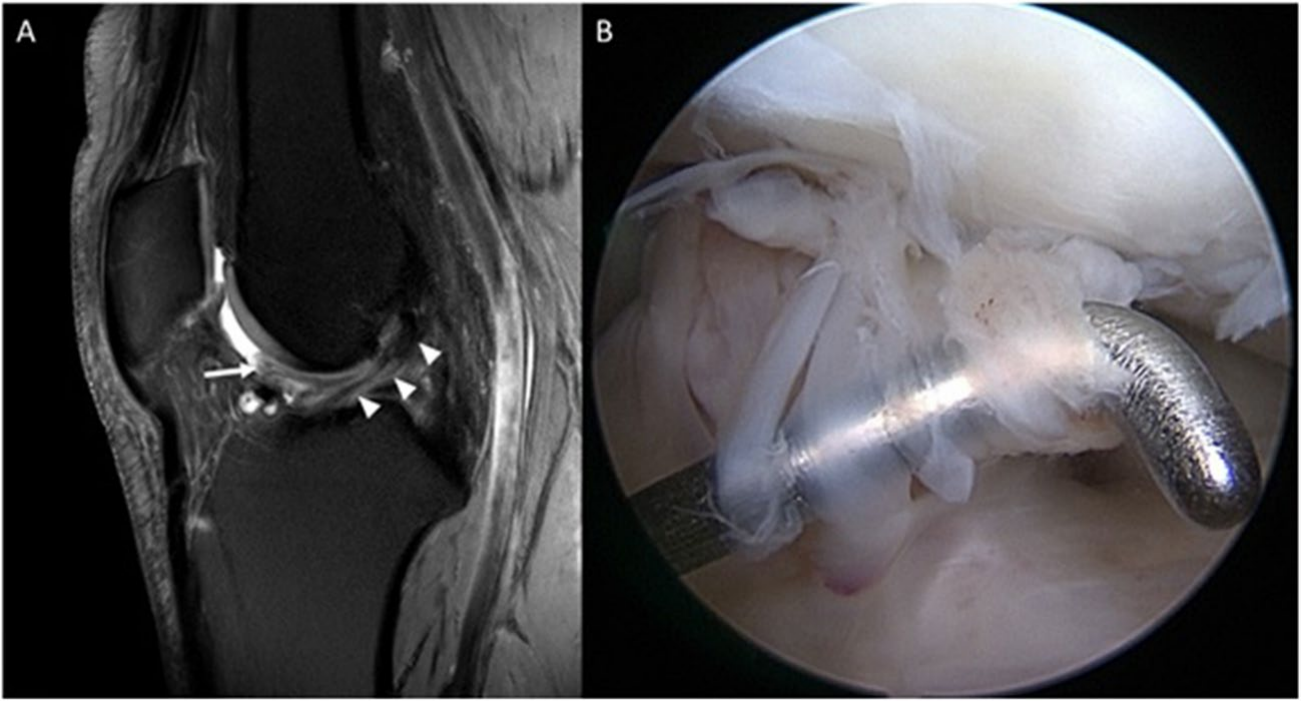
**A** 7T proton density-weighted fat-saturated MRI image of a 24-year-old male patient 1.5 years after primary ACL repair with fraying of the ACL and prolapse of ACL fibers into the intercondylar notch (arrow). The remaining parts of the ACL appear continuous (arrowheads). **B** Corresponding arthroscopic appearance of fraying in the same patient. The repaired ACL itself was continuous and stable

Among the patients, 23 individuals had MR morphologically continuous ACL after 1.5 years, representing 74.2% of the included patients. Fraying was detected in seven patients (22.6%) (Fig. 3, section B). One patient had a non-continuous ACL. In the control group, all 13 subjects (100%) had a continuous ACL, see Table 3, section B.

In the comparison regarding the Lysholm score between the patients with fraying (Lysholm score mean of 91.25 ± 7.96 points) and those with continuous ACL (Lysholm score mean of 90.39 ± 9.75 points), no statistically significant difference occurred (*p* value = 0.76).

The single patient with re-rupture after ACL repair described clinical deterioration, which was also reflected in the Lysholm score. The average Lysholm score of the patients with continuous ACL was 90.27 points; the patient with re-rupture had a Lysholm score of 26 points.

In the assessment of the signal intensity, there was a normal signal intensity in both the sagittal and coronal planes in 30 of the patients and a homogeneously hyperintense signal in one patient. An elongation of the ACL was described in nine patients (29%), whereas no elongation was present in the healthy controls.

#### Quantitative MR assessments—T2/T2* mapping

##### Anterior cruciate ligament

The ACL tissue showed no statistically significant difference when compared regarding the T2 time between ACL repair and ACL of healthy controls, see Table 4. In addition, there was no difference in the comparison between the proximal and distal portions of ACL T2 time after repair (*p *= 0.91).

**Table 4:** T2 values of the ACL in patients and controls

T2 values (ms) ACL	Bracing mean ± SD	Control mean ± SD	*p* value difference
Proximal/cranial	29.09 ± 25.73	30.83 ± 10.74	0.743
Distal/caudal	29.79 ± 32.75	31.47 ± 10.79	0.801

##### Cartilage

T2* values of the ROIs in the femoral and tibial cartilage showed higher values in the ACL repair group, with significant differences in all ROIs except at the posterolateral femur, see Table 5.

**Table 5:** T2* values of the femoral and tibial cartilage in patients and controls

T2* values (ms)	Bracing	Control	
Cartilage			
	Mean ± SD	Mean ± SD	*p* value difference
ROI			
Medial			
Femoral anterior	20.68 ± 6.22	10.95 ± 3.01	< 0.001
Femoral central	25.53 ± 11.52	12.68 ± 3.69	< 0.001
Femoral posterior	28.46 ± 7.16	20.66 ± 6.04	0.003
Tibial anterior	17.66 ± 8.45	11.68 ± 2.78	0.020
Tibial central	18.45 ± 6.16	12.97 ± 2.15	0.004
Tibial posterior	25.53 ± 5.72	13.47 ± 2.79	< 0.001
Lateral			
Femoral anterior	21.91 ± 8.07	12.01 ± 2.94	< 0.001
Femoral central	21.41 ± 4.46	17.79 ± 3.72	0.020
Femoral posterior	27.46 ± 6.71	24.87 ± 4.49	0.228
Tibial anterior	22.68 ± 6.05	13.66 ± 2.52	< 0.001
Tibial central	28.06 ± 9.66	15.41 ± 3.12	< 0.001
Tibial posterior	30.01 ± 8.91	16.27 ± 3.62	< 0.001

### Reliability of qualitative and quantitative results

Inter-reader agreements revealed almost perfect agreement for width (*K *= 0.90), continuity (*K *= 0.85), elongation (*K *= 0.94), and the signal intensity (*K *= 0.94) of the ACL repair. Intrareader agreements of both the radiologist and the orthopedic surgeon also showed almost perfect agreements for width (*K *= 0.90, *K *= 0.84), continuity (*K *= 0.86, *K *= 0.91), elongation (*K *= 1, *K *= 0.94), and signal intensity (*K *= 0.87, *K *= 0.82). Excellent reliability was observed for both T2 values of the ACL repair and T2* values of the cartilage with an ICC of 0.94, respectively with an ICC of 0.89.

## Discussion

Our study demonstrates a good functional outcome 1.5 years after primary arthroscopic ACL repair, whereas the ACL appears often with an increased width in MRI. In 23% of the patients, fraying of the ACL occurred without having any impact in regard to the functional outcome. Quantitative assessment of ACL repair using T2 mapping suggests a similar biochemical tissue composition compared to matched healthy controls. In contrast, quantitative cartilage analyses using T2* mapping indicate early cartilage degradation in patients with ACL repair at the femoral and tibial articular surface.

An improved understanding of the pathophysiology of ligamentous healing has led to increasing interest in treating acute proximal ACL avulsions with primary surgical repair techniques [29], whereas knowledge about the morphological imaging appearance of the ACL and its structural properties after primary repair is very limited. The historical approach of primary ACL repair led to disappointing outcomes [29]. The attempt to overcome the limitations of the past has triggered the development of new surgical techniques [29]. In general, proximal tear patterns have better healing potential than distal or midsubstance tears, with better outcomes in younger patients [30]. In addition, some forms of internal bracing, with either nonabsorbable sutures, scaffolds, or a graft, have been described to increase the success rate of the procedure [30]. The reinforcement of the ACL with internal bracing may protect the ligament during the healing phase, supporting early mobilization, and is thought to encourage natural healing of the ligament [24, 31]. All enrolled patients in our study were treated with arthroscopic suture and internal bracing in acute proximal ACL ruptures by the same surgeon and showed a good functional outcome with a Lysholm score of 87.19 and an IKDC score of 80.23. DiFelice et al described in a case series of 11 patients with proximal ACL rupture and arthroscopic ACL repair with internal bracing a Lysholm score of 93.2 and IKDC score of 86.4 after a mean follow-up time of 3.5 years, which is in line with our study [32]. They reported a treatment failure in one patient, resulting in a failure rate of 9% [32]. One of our patients (3%) also suffered an ACL failure in the sense of a re-rupture caused by a repeated distortion.

MRI is an indispensable tool in the appropriate preoperative and postoperative management of knee ligament injury [27, 33]. A meta-analysis about the diagnostic efficacy of 3T MRI for knee injuries using arthroscopy as a reference standard reported a mean sensitivity of 92% and a mean specificity of 99% for the identification of ACL injuries [34]. In addition, a superior diagnostic efficacy for assessing ACL integrity was described when compared with studies of 1.5T scanners [34]. In general, higher magnetic field strengths provide a higher signal-to-noise ratio, which can be used to increase spatial resolution [15]. In studies comparing knee MRI at 3T and 7T with comparable acquisition times improved overall diagnostic confidence with significantly higher diagnostic accuracy for small joint structures and subtle lesions [35, 36]. However, regardless of the field strength and resolution, knowledge about the imaging appearance after surgical ACL repair is crucial for a correct imaging interpretation. In our study using a 7T MRI a significant increased width of the ACL after repair in the sagittal plane occurred compared to healthy controls (1.04 ± 0.23 cm vs. 0.87 ± 0.15 cm, *p *= 0.026). The ACL itself appeared with normal heterogeneous signal intensity in 96.8% of all our patients at follow-up. Seven patients (22.6%) presented with fraying of the ACL after repair, meaning a loosening of ACL tissue with bulging of ACL fibers into the joint. Knowledge about a potential fraying of the ACL after surgical repair seems to be from outstanding importance for imaging interpretation. There was no statistical difference between patients with fraying compared to patients with continuous ACL repair regarding the Lysholm Score, indicating that the fraying does not necessarily have a clinical relevance. This means that fraying alone, with good clinical examination results, is not necessarily a cause for concern for the patient or surgeon. Herewith, our results join the sparse existing literature about the imaging appearance of the ACL after repair. Ferretti et al reported a normal morphology of the ACL in 10 patients (100%) 6 months after surgical repair, whereas the surgical technique was comparable to our study [37]. The signal intensity of the ACL after repair was rated as isointense in nine of ten patients and intermediate in one of ten patients 6 months after surgery. Possibly morphological alterations (increased width, fraying), as described in our cohort, are not visible 6 months after surgery and develop in the course of time due to continuous or abrupt changes. For other surgical procedures such as ACL reconstruction with hamstring autograft, also a continuous maturation process in the first 2 years after surgery is reported [38].

Besides benefits for morphological imaging, the most striking advantage of ultra-high-field MRI at 7T might be the increased feasibility of performing compositional imaging [17, 19, 22, 39]. Compositional MRI techniques such as T2 and T2* mapping enable noninvasive tissue quantification, providing information about structural changes and tissues’ molecular status [19, 20, 22, 39]. T2 and T2* relaxation times are influenced by the orientation of collagen, collagen content, and tissue hydration and have been used both for ACL and knee cartilage assessment at 7T MRI [20]. Excellent inter-rater and intra-rater reliability regarding T2 values of the ACL in patients with osteoarthritis (OA) and in healthy controls were described before for 3T and 7T MRI, which is in line with our results [20]. The quantitative ACL assessment using T2 mapping in our study revealed no significant difference between patients and matched healthy controls, implying that the tissue composition remains intact. This supports the idea that ACL repair can restore nearly normal knee joint biomechanics.

However, quantitative analysis of femoral and tibial articular cartilage using T2* mapping revealed significantly increased values in our patients with ACL repair compared to healthy controls. Previous examinations of T2* in patients with OA and after ACL injury have shown that T2* values in articular cartilage are typically elevated with increased cartilage degeneration, as well as after ACL reconstruction [40, 41].

There are some limitations to consider. First is the small sample size, with 31 patients and 13 controls. Second is the lack of arthroscopic and histopathological validation of the MRI findings, which is not justifiable for ethical reasons. Third, we only used 7T MRI and did not perform a comparison with other field strengths or with other imaging techniques. Fourth, the lack of histopathological correlation between ACL tissue and articular cartilage, which required an ex vivo approach and was not the intent of the present study. However, comparative assessments of histological and quantitative MRI features have previously been performed, in particular for T2* analysis of the articular cartilage at the knee [40].

## Conclusion

Our study results indicate an identical biochemical tissue composition of the ACL after repair 1.5 years after surgery, whereas the ACL is accompanied by an increased width in 7T MRI imaging. Fraying of the ACL can occur without having any impact in regard to the functional outcome. Knowledge about morphological changes of the ACL after repair may be crucial for a correct imaging interpretation. Although advantages of ACL repair over reconstruction techniques are anticipated in regard to restoring native anatomic tissue and preserving proprioception T2* analysis indicates early cartilage degradation in our patients. Further prospective multicenter randomized controlled trials are warranted to elucidate repair versus reconstruction for proximal anterior cruciate ligament tears.

## Abbreviations

ACL: Anterior Cruciate Ligament
ICC: Intraclass correlation coefficient
IKDC: International Knee Documentation Committee
PD-fs: Proton density-weighted images with fat saturation
ROM: Range of motion
SD: Standard deviation
SNR: Signal-to-noise ratio

## Declarations

### Guarantor

The scientific guarantor of this publication is Milena L. Pachowsky.

### Statistics and biometry

One of the authors has significant statistical expertise. However, no complex statistical methods were necessary for this paper.

### Study subjects or cohorts overlap

No study subjects or cohorts have been previously reported.

### Methodology

• prospective

• experimental

• performed at one institution

## References

[1] Hewett TE, Di Stasi SL, Myer GD (2013). Current concepts for injury prevention in athletes after anterior cruciate ligament reconstruction. Am J Sports Med.

[2] Sanders TL, Maradit Kremers H, Bryan AJ (2016). Incidence of and factors associated with the decision to undergo anterior cruciate ligament reconstruction 1 to 10 years after injury. Am J Sports Med.

[3] Sanders TL, Maradit Kremers H, Bryan AJ (2016). Incidence of anterior cruciate ligament tears and reconstruction: a 21-year population-based study. Am J Sports Med.

[4] van der List JP, DiFelice GS (2017). Primary repair of the anterior cruciate ligament: a paradigm shift. Surgeon.

[5] van der List JP, DiFelice GS (2017). Role of tear location on outcomes of open primary repair of the anterior cruciate ligament: a systematic review of historical studies. Knee.

[6] Filbay SR, Roemer FW, Lohmander LS (2023). Evidence of ACL healing on MRI following ACL rupture treated with rehabilitation alone may be associated with better patient-reported outcomes: a secondary analysis from the KANON trial. Br J Sports Med.

[7] Barrett DS (1991). Proprioception and function after anterior cruciate reconstruction. J Bone Joint Surg Br.

[8] Dhillon MS, Bali K, Prabhakar S (2012). Differences among mechanoreceptors in healthy and injured anterior cruciate ligaments and their clinical importance. Muscles Ligaments Tendons J.

[9] van der List JP, DiFelice GS (2017). Range of motion and complications following primary repair versus reconstruction of the anterior cruciate ligament. Knee.

[10] Toy BJ, Yeasting RA, Morse DE, McCann P (1995). Arterial supply to the human anterior cruciate ligament. J Athl Train.

[11] Nguyen DT, Ramwadhdoebe TH, van der Hart CP, Blankevoort L, Tak PP, van Dijk CN (2014). Intrinsic healing response of the human anterior cruciate ligament: an histological study of reattached ACL remnants. J Orthop Res.

[12] Figueroa D, Melean P, Calvo R (2010). Magnetic resonance imaging evaluation of the integration and maturation of semitendinosus-gracilis graft in anterior cruciate ligament reconstruction using autologous platelet concentrate. Arthroscopy.

[13] Hashemi J, Mansouri H, Chandrashekar N, Slauterbeck JR, Hardy DM, Beynnon BD (2011). Age, sex, body anthropometry, and ACL size predict the structural properties of the human anterior cruciate ligament. J Orthop Res.

[14] Radice F, Yanez R, Gutierrez V, Rosales J, Pinedo M, Coda S (2010). Comparison of magnetic resonance imaging findings in anterior cruciate ligament grafts with and without autologous platelet-derived growth factors. Arthroscopy.

[15] Krug R, Stehling C, Kelley DA, Majumdar S, Link TM (2009). Imaging of the musculoskeletal system in vivo using ultra-high field magnetic resonance at 7 T. Invest Radiol.

[16] Ladd ME, Bachert P, Meyerspeer M (2018). Pros and cons of ultra-high-field MRI/MRS for human application. Prog Nucl Magn Reson Spectrosc.

[17] Welsch GH, Juras V, Szomolanyi P (2012). Magnetic resonance imaging of the knee at 3 and 7 tesla: a comparison using dedicated multi-channel coils and optimised 2D and 3D protocols. Eur Radiol.

[18] Tao H, Qiao Y, Hu Y (2018). Quantitative T2-mapping and T2()-mapping evaluation of changes in cartilage matrix after acute anterior cruciate ligament rupture and the correlation between the results of both methods. Biomed Res Int.

[19] Hesper T, Hosalkar HS, Bittersohl D (2014). T2* mapping for articular cartilage assessment: principles, current applications, and future prospects. Skeletal Radiol.

[20] Anz AW, Edison J, Denney TS (2020). 3-T MRI mapping is a valid in vivo method of quantitatively evaluating the anterior cruciate ligament: rater reliability and comparison across age. Skeletal Radiol.

[21] Ranmuthu CDS, MacKay JW, Crowe VA, Kaggie JD, Kessler DA, McDonnell SM (2021). Quantitative analysis of the ACL and PCL using T1rho and T2 relaxation time mapping: an exploratory, cross-sectional comparison between OA and healthy control knees. BMC Musculoskelet Disord.

[22] Newbould RD, Miller SR, Toms LD (2012). T2* measurement of the knee articular cartilage in osteoarthritis at 3T. J Magn Reson Imaging.

[23] Baum T, Joseph GB, Karampinos DC, Jungmann PM, Link TM, Bauer JS (2013). Cartilage and meniscal T2 relaxation time as noninvasive biomarker for knee osteoarthritis and cartilage repair procedures. Osteoarthritis Cartilage.

[24] van der List JP, DiFelice GS (2017). Arthroscopic primary anterior cruciate ligament repair with suture augmentation. Arthrosc Tech.

[25] Lysholm J, Gillquist J (1982). Evaluation of knee ligament surgery results with special emphasis on use of a scoring scale. Am J Sports Med.

[26] Hefti F, Muller W, Jakob RP, Staubli HU (1993). Evaluation of knee ligament injuries with the IKDC form. Knee Surg Sports Traumatol Arthrosc.

[27] Naraghi AM, White LM (2016). Imaging of athletic injuries of knee ligaments and menisci: sports imaging series. Radiology.

[28] Schmitt B, Zbyn S, Stelzeneder D (2011). Cartilage quality assessment by using glycosaminoglycan chemical exchange saturation transfer and (23)Na MR imaging at 7 T. Radiology.

[29] Batista JP, Chahla J, Dalmau-Pastor M, Maestu R, Kunze KN, Guelfi M (2021). Arthroscopic anterior cruciate ligament repair with and without suture augmentation: technical note. J ISAKOS.

[30] van Eck CF, Limpisvasti O, ElAttrache NS (2018). Is there a role for internal bracing and repair of the anterior cruciate ligament? A systematic literature review. Am J Sports Med.

[31] Bachmaier S, DiFelice GS, Sonnery-Cottet B (2020). Treatment of acute proximal anterior cruciate ligament tears-Part 2: the role of internal bracing on gap formation and stabilization of repair techniques. Orthop J Sports Med.

[32] DiFelice GS, Villegas C, Taylor S (2015). Anterior cruciate ligament preservation: early results of a novel arthroscopic technique for suture anchor primary anterior cruciate ligament repair. Arthroscopy.

[33] Geeslin AG, Lemos DF, Geeslin MG (2021). Knee ligament imaging: preoperative and postoperative evaluation. Clin Sports Med.

[34] Smith C, McGarvey C, Harb Z (2016). Diagnostic efficacy of 3-T MRI for knee injuries using arthroscopy as a reference standard: a meta-analysis. AJR Am J Roentgenol.

[35] Friebe B, Richter M, Penzlin S (2018). Assessment of low-grade meniscal and cartilage damage of the knee at 7 T: a comparison to 3 T imaging with arthroscopic correlation. Invest Radiol.

[36] Springer E, Bohndorf K, Juras V (2017). Comparison of routine knee magnetic resonance imaging at 3 T and 7 T. Invest Radiol.

[37] Ferretti A, Monaco E, Annibaldi A (2020). The healing potential of an acutely repaired ACL: a sequential MRI study. J Orthop Traumatol.

[38] Lutz PM, Achtnich A, Schutte V, Woertler K, Imhoff AB, Willinger L (2022). Anterior cruciate ligament autograft maturation on sequential postoperative MRI is not correlated with clinical outcome and anterior knee stability. Knee Surg Sports Traumatol Arthrosc.

[39] Mosher TJ, Dardzinski BJ (2004). Cartilage MRI T2 relaxation time mapping: overview and applications. Semin Musculoskelet Radiol.

[40] Williams A, Qian Y, Bear D, Chu CR (2010). Assessing degeneration of human articular cartilage with ultra-short echo time (UTE) T2* mapping. Osteoarthritis Cartilage.

[41] Williams AA, Erhart-Hledik JC, Asay JL (2021). Patient-reported outcomes and knee mechanics correlate with patellofemoral deep cartilage UTE-T2* 2 years after anterior cruciate ligament reconstruction. Am J Sports Med.

